# Once bitten, twice shy? Lessons learned from an experiment to liberalize price regulations for dental care

**DOI:** 10.1007/s10198-019-01145-z

**Published:** 2019-12-31

**Authors:** Anna-Lena Trescher, Stefan Listl, Onno van der Galien, ADVOCATE Consortium, Frank Gabel, Olivier Kalmus

**Affiliations:** 1 Department of Conservative Dentistry - Section for Translational Health Economics, Heidelberg University Hospital, Im Neuenheimer Feld 400, 69120 Heidelberg, Germany; 2 Department of Dentistry - Quality and Safety of Oral Healthcare, Radboud University Medical Center, Radboud Institute for Health Sciences, Philips van Leydenlaan 25, 6525 EX Nijmegen, The Netherlands; 3 Zilveren Kruis Achmea, Storkstraat 12, 3833 LB Leusden, The Netherlands

**Keywords:** Price liberalization, Quasi-experimental design, Prevention, Oral care, I11, I18

## Abstract

In 2012, The Netherlands established the so-called “free market experiment”, which allowed providers of dental care to set the prices for their dental services themselves. The introduction of market mechanisms is intended to improve the quality of care and to contribute to cost containment, but increasing health expenditures for citizens have been observed in this context. Using large-volume health insurance claims data and exploiting the 2012 experiment in Dutch dental care, we identified the effects of a liberalization of service prices. Using pooled regression with individual fixed effects, we analyzed changes in utilization patterns of prevention-oriented dental services in response to the experiment as well as the elasticities in demand in response to variations in out-of-pocket (OOP) prices. We found substantial increases in prices and patients’ OOP contributions for dental services following the liberalization with differences in increases between types of services. In response to the experiment, the proportion of treatment sessions containing preventive-oriented services decreased significantly by 3.4% among adults and by 5.3% for children and adolescents. Estimates of short-run price elasticities of demand for different services point towards differences in price sensitivity. One potential explanation for the observed variations in prices and utilization could be different extents of asymmetric information for first-stage and follow-on services. Price liberalization seems to have affected the composition of treatment sessions towards a decreasing use of preventive services, suggesting a shift in the reason for seeing a dental care provider from a regular-preventive perspective to a symptom-based restorative approach.

**Electronic supplementary material:**

The online version of this article (10.1007/s10198-019-01145-z) contains supplementary material, which is available to authorized users.

## Background

Deciphering the market mechanisms in health care is a long-standing and highly relevant matter of debate in health policy and health economic research [1]. There is an abundance of theoretical frameworks highlighting the specific characteristics of health care markets (uncertainties, externalities, and asymmetric information) and the relevance of market regulation in health care. However, studies empirically observing the impacts of deregulating interventions remain scarce.

Throughout recent years and decades, a number of European countries have liberalized their health care systems to various degrees [2, 3]. The introduction of market mechanisms is intended to improve the quality of care and to contribute to cost containment. However, increasing health expenditures for citizens have been observed as a result of such liberalizations [4]. Whereas comprehensive insurance coverage often mitigates the effects of higher prices for care relating to more life-threatening health conditions, dental care is somewhat different as dental services are frequently subject to a higher proportion of the expenditures being paid by the patients [5]. Price changes in dental care may, therefore, lead to substantial variations in utilization of dental care. Although this could in turn influence people’s oral health, little is known about the effects of deregulation and market liberalization on out-of-pocket (OOP) prices, price elasticities, and utilization of dental care.

A movement towards more market mechanisms in oral health care can generally be promoted in various ways: for instance, by excluding oral health care coverage from public insurance schemes or by deregulating price mechanisms. Whereas the former has a direct effect on patients OOP payments when seeking care, the effects of the latter are less straightforward and depend on market characteristics.

Health economic theory suggests that health care markets differ from those of other goods in substantial ways [1]. Moreover, health care includes various different types of services whose market mechanisms may function differently when compared to each other [6]. In particular, health care services can be grouped into informative first-stage services, such as examinations and diagnostics and second-stage follow-on services that consist of any subsequent treatment [7]. Demand for the latter kind of services is dependent on and determined by consumption of the former. It could be argued that consumers may be informed better about the first-stage services, as they are consumed more frequently and can be considered to be often less technically complex. Along these lines, demand could be more price sensitive for such services [8]. This means that, in theory, competition should affect prices for dental diagnostics and examinations to a higher degree than prices for invasive and restorative follow-on services [7].

Competition on health care markets can be limited through supply-side market power [9]. Hence, if dentists can exploit their information surpluses, price liberalization for dental care could lead to price increases. Information asymmetries between dental care providers and patients are likely more pronounced for complex follow-on services than for the first-stage services (e.g., in terms of insight in the need for specific services, their potential effectiveness, and the quality of care). The presence of supply-sided market power is supported both by theory and empirical evidence [10]. A lack of transparency about dental services presented in a long list of detailed codes complicate comparisons between individual dental care providers and limit the information of the individual consumer. Patients also tend to establish longer term treatment relationships with and trust in their dentists which can be considered to imply increasing transaction costs when changing the dentist. Such effects can be particularly pronounced for complex invasive services [5]. Moreover, for the country examined in our study (the Netherlands), previous evidence points towards a relatively small number of and market access barriers for dental care providers, potentially leading to supply-sided market power [11].

To date, the empirical evidence on the effect of competition on prices for dental care services remains sparse. Norway deregulated its fee system for adult dental care in 1995 [12]. This replaced a system with fixed fees determined by annual negotiations between governmental bodies and provider representatives. In the short run, this price competition only led to small variations among the fees which dentists charged. The authors concluded that dentists did not seem to exploit their market power, since their action was constrained by motives other than self-interest; however, they also conceded that dentists and patients might not yet have adjusted to the changes in the short time. In Sweden, prices for dental care are also market-determined. Chirico 2013, applying her own model, showed that the effect of the current-level competition on prices in the Swedish system is negative, but small. Simulations run on the Swedish data show room for further price decreases, especially for the first-stage services.

Health economic evidence suggests that dental care utilization is significantly determined by the OOP service price [13, 14]. For a Finnish population, it was shown that OOP prices had a small but significant effect on utilization, both on the probability of visiting a dentist and the amount of care consumed [15]. Little or no evidence exists on how the composition of dental care demand changed with altering OOP prices. From a theoretical perspective, demand for preventive services that function as self-protection should increase if OOP prices for follow-on services increase [16]. On the other hand, evidence from general health care shows undesired “offsets” towards greater use and spending on other services such as acute care in reaction to increases in cost sharing for preventive/chronic care [17–19]. Such unintended effects could also be of relevance for dental care, since preventive services, such as fluoride varnish or sealants, are effective measures to prevent oral disease [20, 21]. Therefore, many dental insurance schemes have no co-payments for routine preventive services in contrast to other dental services [5]. However, if increases of OOP prices for preventive dental care are disproportionally larger than increases of OOP prices for other dental services, this may change patients’ motives for seeing a dental care provider from a regular-prevention-oriented approach to symptom-based visits.

The price elasticity of demand overall for dental services is relatively inelastic. This means that a specific percentage increase in prices leads to a relatively lower percentage decrease in demand. Evidence also suggests that price elasticities for dental care are smaller in groups with insurance coverage for dental care compared to that of non-insured individuals. In insured populations, estimates on OOP price elasticities for dental services range from 0 to − 0.47 [22–24]. Based on a sample of Finnish employees, Sintonen and Maljanen [15] estimated an OOP price elasticity of − 0.069 for dental care. Hence, evidence suggests that changes in demand for dental care are rather small in reaction to price changes.

To our knowledge, only two studies have estimated price elasticities of demand for specific dental services: Manning and Phelps [13] estimated significant price elasticities of demand for preventive and check-up services between − 0.56 and − 1.34 and between − 1.51 and 0.58 for restorative care. Here, no clear pattern of differentiating price elasticities of demand was observable. In contrast, price elasticities of demand estimated by Meyerhoefer et al. [13] were not significant and close to zero for preventive, basic restorative, and extensive restorative services.

In 2012, the Netherlands embarked on the so-called “free market experiment” (FME) which enabled dental care providers to set the prices for their dental services themselves. The previous price ceiling for dental fees was temporarily abolished. In this paper, we exploit this natural experiment with respect to the market structure of dental care to identify, by means of large-scale administrative data, the impacts of market liberalization on dental care. The current study has three primary goals. First, analyze short-run OOP price developments. Different services are analyzed separately to take into account market differences between the first-stage and second-stage services. Second, the study analyzes the impact of price liberalization on relative utilization of dental care services. Finally, we calculate short-run price elasticities of demand for specific dental care services. To our knowledge, this is the first study to look into these effects for individual dental care services, in particular preventive services compared to restorative and invasive services.

After this introduction, the remainder of the paper is organized as follows: “Institutional background: the Dutch-free market experiment” provides an overview of the reform under evaluation. In the section “Methods”, we describe the data set, which required data harmonization and econometric methods applied. The next section presents the results and the final section provides a discussion.

## Institutional background: the Dutch-free market experiment

In 2006, a major health care reform introduced a system of managed competition in the health insurance market and initiated implementation of competition in health care purchasing and delivery [25]. In this process of establishing market mechanisms, the introduction of price liberalization in dental care was also subject of political debate [26]. In 2011, the government decided to introduce such liberalizations in the dental care market on an experimental basis [27]. Besides cost containment and the improvement of service quality, the experiment’s main objective was to promote entrepreneurship and to provide incentives for the dissemination of product innovations in the market [28]. The experiment should provide the opportunity to examine how prices, quality, and accessibility developed in a liberalized market.

While dental care is provided under the basic insurance coverage for children and adolescents in the Netherlands, the majority of adult dental services are not covered by this basic package. Additionally, the deductible of the basic health insurance as well as specific co-payments apply to those adult dental services covered by the basic insurance package. 88% of the insured population purchased additional insurance coverage in 2012 and 76% of these contracts also covered dental care [29]. This meant that two-thirds of the adult population took out additional insurance coverage for dental care in the reform year.

Dental care is reimbursed on a fee-for-service based system in the Netherlands both for services covered through the basic health insurance, as well as those covered through co-insurance. Prior to the experiment, a price ceiling existed for dental services determining a maximum fee for each dental service item. The experiment introduced on January 1, 2012 allowed dental care providers to set the prices for their dental services on their own.

This price liberalization for dental services was accompanied by a major overhaul of the fee schedule [30]. To promote service transparency and provide better comparability for patients, the 400 items of the existing fee schedule were summarized into 150 more comprehensible, wider diagnostic and treatment codes. To maintain price transparency, dental care providers had to make their price lists openly available for the patients, for example in the waiting rooms and on their websites [28]. As there was no document offering a translation of the new codes to the previous ones, a price comparison to the pre-intervention period was not possible, however.

Although the experiment was initially planned to last for 3 years, it was terminated at the end of 2012. This was mainly due to observed increases in service prices. Analyses of market prices for a small basket of services shortly after the start of the experiment indicated a 4% increase in prices for dental services [31]. Subsequent analyses of price changes following the price liberalization showed heterogeneous effects. Repeated official reports by the Dutch Healthcare Authority (Nederlandse Zorgautoriteit: NZa) concluded price increases by 9.6% and 10.7% based on the comparison of a subgroup of service items [32, 33]. In contrast, a report commissioned by the Dutch Dentist Association (ANT) found a price increase of 3% attributable to the price liberalization [34]. They claimed that the main proportion of the increase observed by NZa’s official report traced back to the extensive rearrangement of the fee schedule.

The three biggest health insurance companies in the Netherlands defined maximum reimbursement rates for dental services in 2012, the year of the FME [35]. These rates applied to children covered under the basic insurance and adults covered by dental co-insurance. The fraction of the fee that exceeded maximum compensation rates had to be paid out-of-pocket by the patient. Therefore, higher prices translated into higher OOP expenditures for patients. Following the reform, people also faced potential OOP expenditures for the treatment of their children, which had been basically free before. The insurance company Achmea increased these rates during the year, as they were realizing that their customers were facing increasing OOP expenditures [36].

## Methods

### Data

We obtained anonymized claim-level administrative data spanning a 3-year period from 2011 to 2013 from the Achmea Health Database.^1^ The data set included all services that were provided to Achmea customers within this period. These were patients with additional dental insurance as well as children and adolescents having received care under basic health insurance coverage. The data set provided information about procedure codes, the calendar week of service provision, unique patient and provider identifiers, patients’ age groups (in 5-year intervals), patients’ sexes and insurance statuses, as well as complete cost information. Cost data included provider fee and insurance reimbursements. The data also included the deductibles and co-payments for dental services covered by the basic health insurance. In total, the data set comprised 41,145,107 claims from 19,542,857 treatment sessions for 3,118,517 unique patients. Services were mainly performed by dentists. Only 10% of claims were made by dental hygienist. A patient treatment session is defined as “all treatments provided to a patient within one week”.

### Data harmonization

We faced the same challenge as previous analyses of the 2012 free market experiment [32–34], which was an adequate harmonization of treatment codes. Although procedure codes changed substantially in 2012, the NZa did not release a translation scheme. To enable a comparison of services provided over several years, we harmonized procedure codes from 2011 to 2013 into service baskets (see Table 1) [37–40]. A detailed overview is provided in the Appendix Table A1. Due to substantial changes in procedure codes, it was not possible to harmonize all services. Besides preventive services, we selected three additional treatment/diagnostic items to enable comparisons to be made. Additionally, the changes in the fee schedule made it impossible to analyze changes in overall dental care service utilization.

**Table 1:** Service baskets and associated procedure codes

Service baskets	Procedure codes
Preventive examinations and oral hygiene advice	C11, C12, M31, M70; A111, C112, C114, C124; C11, C13, M01, M02
Scaling and polishing	M50, M55, M59; C212, C214; M03
Fissure sealants (only for children/adolescents)	V30, V35; C511; V30, V35
Fluoride applications (only for children/adolescents)	M10, M20, M21; C611, C811; M05, M10, M20
Direct restorations	V10, V11, V12, V13, V14 (V20, V21, V50, V60, V70, V80, V85); E111, E112, E113, E114, E131, E411; V11, V12, V13, V14 (V20, V21, V50, V60, V70, V80, V85)
Extractions	H10, H15, H30, H35 (H20, H21, H25, H90); J311, J315; H35, H11, H16 (H21, H90)
Radiographs	X10, X21, X22, X24; A311, A321, A324, A327; X10, X21, X22, X24, X25, X26

For each of these groups of services, a binary variable was set to 1 for a particular treatment session if at least one corresponding code was remunerated in that session. Otherwise, it was set to 0. On a session level, we totaled payment information per service basket to obtain one value for every payment variable of interest for each basket.

### Empirical strategy

Short-term changes in dental care fees are of interest for people without insurance coverage for dental care. However, for those people with coverage for dental services, changes in OOP payments are a relevant decision criterion determining their utilization of oral health care. Patient OOP expenditures were calculated as the difference between the declared dentist fee and insurance reimbursement received by the patients for each patient and each selected service.

To identify the experiment’s impact on the relative utilization of dental care services, we compared utilization of services in the pre-experiment year and in the year of the experiment. The analyses estimate the likelihood that a treatment session included the treatment item of interest. Analysis does not allow statements of changes in overall dental care use, but evaluates shifts in the composition of utilization. On an individual treatment session level, we applied pooled panel logistic regression. We accounted for substantial volatility of utilization over the calendar year, by restricting the analysis to two calendar years with one pre-introduction and one post-introduction and by pooling data in the pre- and post-introduction period to reduce variance. To account for unobserved but fixed individual patient characteristics, we exploited the longitudinal character of the underlying data and included patient-level fixed effects [41]:

1 $$Y_{it} \; = \;\alpha_{i} \; + \;\beta \;\left( {\text{Re} {\text{form}}_{it} } \right)\; + \;\varepsilon_{it} .$$

We performed regression analyses according to Eq. (1) for each service basket. $Y_{it}$—the variable of interest—is a dummy variable for each treatment session of patient $i$ at time $t$ indicating whether it includes at least one procedure of the particular service basket or not. ${\text{Reform}}_{it}$ denotes a dummy variable indicating the reform period. Hereby, $\beta$ is our estimator of interest. $\varepsilon_{it}$ represents the error term. Following previous analyses, indicating that reactions in utilization may considerably differ between children and adults, we stratified our analyses for children and adolescents up to the age of 18 years and adults. As a robustness check, we repeated the analyses comparing the year of the reform with the post-reform year, where fixed fee-for-service remuneration was re-introduced. These results are presented in Appendix Tables A4 and A5.

To combine information about OOP prices and service utilization, we calculated OOP price elasticities of demand for every service under evaluation. This reflected the reaction of demand on price changes in the short run. We exploited the variability in prices and utilization induced by the reform. In the context of this short-run perspective under evaluation, we treated the price information as an exogenous variable. We assumed that in the short-time duration of the experiment, dental care providers used their pricing power in an environment with fixed and limited supply. Observed utilization of the specific service was used as a proxy for demand [14]. We aggregated data at an annual provider level giving us the number of services provided and average prices per provider per year for every service basket. To obtain a wider range of price and utilization information, we included observations from 2011 to 2013 in the analysis. We performed Poisson regression due to the count data character of the outcome variable. Provider-level fixed effects were included to control for unobserved characteristics of the provider, such as practice size, etc. We again stratified for two age groups in this analysis:

2 $$Y_{\text{pt}} \; = \;\alpha_{\text{p}} \; + \;\beta \left( {C_{\text{pt}} } \right)\; + \;\gamma \left( {X_{\text{pt}} } \right)\; + \;\varepsilon_{\text{pt}} .$$

The outcome variable $Y_{pt}$ counts the number of treatment sessions with the respective treatment provided by provider $p$ at time $t$; $C_{\text{pt}}$ represents average patients OOP contribution for this service for provider ${\text{p}}$ at time $t$.$X_{\text{pt}}$ is a vector of control variables containing information about the patient’s age, sex, and additional insurance coverage. $\varepsilon_{\text{pt}}$ denotes the error term. Based on the regression results from Eq. (2), margins at mean were calculated to obtain elasticity estimates. It denotes the proportionate change in demand given a proportionate change in price.

## Results

Table 2 shows summary statistics for dependent and independent variables for the whole sample stratified by age groups and years. The mean of patients’ age was 10 years for children and adolescents and 46 years for adults. Preventive services were utilized in most sessions in both age groups. As a result, preventive examinations and oral hygiene advice were most prevalent followed by scaling and polishing for adults. Fissure sealants were placed in 5.9% of treatment sessions for children and adolescents. Turning to the non-preventive items: direct restorations were provided in one-quarter of adult treatment sessions, followed by radiographs. Direct restorations were also the most prevalent service in the younger age group followed by radiographs. Extractions played only a minor role with around 3% in both age groups. Descriptive statistics showed variations between the year of the experiment and the pre- and post-experiment years. The proportion of sessions which included any preventive service dropped in the intervention year. This development was also observed for most preventive items except for scaling and polishing in adults and fluoride applications for children and adolescents. Regarding restorative items and radiographs, descriptive statistics showed mixed developments. The share of session including radiographs for the younger age group slightly increased in the year of the intervention and so did the share of extractions in the adult population. Relative utilization of the remaining items increased in the year of the experiment.

**Table 2:** Summary statistics: dependent and independent variables

	Age group 0–17 mean (SD)			Age group 18+ mean (SD)		
	2011	2012 (intervention)	2013	1011	2012 (intervention)	2013
Patient’s age	10.05 (4.083)	10.08 (4.083)	10.08 (4.094)	46.17 (16.00)	46.44 (16.18)	46.75 (16.27)
Women/girls (% of all patients)	0.490 (0.500)	0.489 (0.500)	0.489 (0.499)	0.540 (0.498)	0.540 (0.498)	0.539 (0.498)
Proportion of sessions including the following intervention						
Prev. exams and oral hygiene advice	0.516 (0.500)	0.481 (0.500)	0.520 (0.500)	0.556 (0.497)	0.516 (0.500)	0.563 (0.496)
Scaling and polishing	0.178 (0.382)	0.149 (0.356)	0.179 (0.383)	0.361 (0.480)	0.368 (0.482)	0.392 (0.488)
Fissure sealants	0.062 (0.240)	0.058 (0.234)	0.057 (0.231)	NA	NA	NA
Fluoride applications	0.222 (0.416)	0.225 (0.417)	0.228 (0.420)	NA	NA	NA
Any preventive service	0.569 (0.495)	0.536 (0.499)	0.573 (0.495)	0.621 (0.485)	0.591 (0.492)	0.660 (0.474)
Direct restorations	0.110 (0.313)	0.099 (0.299)	0.104 (0.305)	0.259 (0.438)	0.248 (0.432)	0.257 (0.437)
Extractions	0.027 (0.16)	0.026 (0.159)	0.026 (0.158)	0.030 (0.172)	0.032 (0.175)	0.026 (0.159)
Radiographs	0.079 (0.270)	0.083 (0.276)	0.079 (0.270)	0.211 (0.408)	0.204 (0.403)	0.212 (0.408)
*N* (number of sessions)	2,027,500	2,032,386	2,179,415	4,503,781	4,395,245	4,404,548

We observed average service fee increases in the reform year for nearly all treatment items under investigation; except for fluoride applications for adult patients (see Appendix Tables A2 and A3). However, the magnitude of increases differed substantially and ranged from 1.5% for radiographs for adults to 45% for tooth extractions for children and adolescents. In general, increases in service fees for children and adolescents were higher than for adults. After the reform was terminated, most service fees dropped, but were still above the pre-reform level. Patients’ OOP expenditures were a multiple of the pre-reform expenditures in 2012 for most of the service items (Tables 3 and 4). Services for children and adolescents come along with substantial co-payments in the year of the experiment, whereas OOP expenditures for these services were previously 0. In 2013, average OOP expenditures nearly returned to the pre-reform baseline values. Regarding first-stage and follow-on services, we observed larger increases in service fees and OOP expenditures for restorative services, such as extractions, than for regular services, such as preventive exams and oral hygiene advices.

**Table 3:** Descriptive statistics: patients’ OOP expenditures for age group 18+

	Average OOP expenditures (SD)			Absolute change in OOP expenditures	
	2011	2012	2013	2012	2013
Prev. exams and oral hygiene advice	0.06 € (0.85)	0.89 € (2.37)	0.06 € (0.99)	+ 0.83 €	− 0.83 €
Scaling and polishing	0.19 € (2.20)	3.00 € (4.45)	0.43 € (4.29)	+ 2.81 €	− 2.57 €
Direct restorations	5.89 € (12.74)	12.93 € (21.25)	5.89 € (12.83)	+ 7.04 €	− 7.04 €
Extractions	4.40 € (10.92)	12.75 € (23.96)	4.64 € (10.75)	+ 8.35 €	− 8.11 €
Radiographs	7.40 € (5.95)	8.48 € (6.81)	7.74 € (6.08)	+ 1.08 €	− 0.74 €

**Table 4:** Descriptive statistics: patients’ OOP expenditures for age group 0–17

	Average OOP expenditures (SD)			Absolute change in OOP expenditures	
	2011	2012	2013	2012	2013
Prev. exams and oral hygiene advice	0.00 € (0.10)	0.25 € (1.42)	0.02 € (19.32)	+ 0.25 €	− 0.23 €
Scaling and polishing	0.01 € (0.27)	0.85 € (2.32)	0.02 € (0.60)	+ 0.84 €	− 0.83€
Fissure sealants	0.01 € (0.67)	2.46 € (8.15)	0.01 € (0.26)	+ 2.45 €	− 2.45 €
Fluoride applications	0.00 € (0.11)	0.46 € (1.07)	0.00 € (0.06)	+ 0,46 €	− 0.46 €
Direct restorations	0.01 € (0.45)	2.01 € (7.88)	0.01 € (0.72)	+ 2.00 €	− 2.00 €
Extractions	0.00 € (0.09)	2.38 € (8.17)	0.01 € (0.30)	+ 2.38 €	− 2.37 €
Radiographs	0.00 € (0.26)	0.97 € (4.24)	0.01 € (0.63)	+ 0.97 €	− 0.96 €

Tables 5, 6 provide logistic regression results with patient fixed effects for utilization changes following the introduction of the experiment. Results are presented as odds ratios and associated percentage changes in utilization. Odds ratios (OR) indicate a decrease in utilization following the reform (OR < 1) or an increase following the reform (OR > 1). All estimates are statistically significant under *α* = 0.01. For patients aged 18 years and older, we observed a significant relative decrease in utilization of preventive services in comparison to overall dental care use: The share of sessions including any preventive service decreased by 3.39% (OR 0.873 [95% CI 0.870–0.876]). However, for specific preventive items, the results were heterogeneous: large decreases were observed for preventive exams and oral hygiene advice (− 4.14%; OR 0.847 [95% CI 0.844; 0.850]), while sessions were more likely to include scaling and polishing (0.48%; OR 1.019 [95% CI 1.016; 1.023]). We also observed significant decreases in the share of direct restorations and radiographs. The fraction of sessions containing extractions increased in the adult population by 1.80% (OR 1.075 [95% CI 1.065; 1.085]) following the reform. For children and adolescents, logistic regression results showed a larger relative decrease in utilization of preventive items compared to the adult population. The fraction of sessions containing any preventive service decreased by 5.29% (OR 0.809 [95% CI 0.804; 0.813]) following the reform. Except for fluoride applications (2.07%, OR 1.086 [95% CI 1.079; 1.093]), the likelihood that treatment sessions included preventive services decreased. The largest decrease was observed for preventive exams and oral hygiene instruction with − 5.30% (OR 0.808 [95% CI 0.804; 0.813]). In comparison to overall dental care use, a scaling and polishing and fissure sealants declined by 2.27% (OR 0.913 [95% CI 0.906; 0.920]) and 2.00% (OR 0.923 [95% CI 0.914; 0.932]) respectively. For non-preventive items, sessions were less likely to include direct restorations (− 1.71%; OR 0.934 [95% CI 0.926; 0.942]) and extractions (− 5.4%; OR 0.804 [95% CI 0.792; 0.816]) in the age group under 18 but more likely to include radiographs (4.08%; OR 1.178 [95% CI 1.168; 1.188]).

**Table 5:** Logistic regression with patient fixed effects for age group 18+

Intervention	Odds ratio during vs. before reform [95% CI]	Associated percentage change (%)	Observations
Prev. exams and oral hygiene advice	0.847 [0.844; 0.850]	− 4.14	7,171,833
Scaling and polishing	1.019 [1.016; 1.023]	0.48	5,781,013
Any preventive service	0.873 [0.870; 0.876]	− 3.39	6,693,394
Direct restorations	0.921 [0.918; 0.924]	− 2.06	6,095,415
Extractions	1.075 [1.065; 1.085]	1.80	1,287,825
Radiographs	0.887 [0.884; 0.890]	− 3.00	6,102,535

Dependent variable: dummy variable for each treatment session of patient *i* at time *t* indicating whether it includes at least one procedure of the particular service basket or not. Model includes patient-level fixed effects. Confidence intervals are denoted in brackets

**Table 6:** Logistic regression with patient fixed effects for age group 0–17

Intervention	Odds ratio during vs. before reform [95% CI]	Associated percentage change (%)	Observations
Prev. exams and oral hygiene advice	0.808 [0.804; 0.813]	− 5.3	3,174,717
Scaling and polishing	0.913 [0.906; 0.920]	− 2.27	2,073,063
Fissure sealants	0.923 [0.914; 0.932]	− 2.00	1,286,610
Fluoride applications	1.086 [1.079;1.093]	2.07	2,319,682
Any preventive service	0.809 [0.804;0.813]	− 5.29	2,934,764
Direct restorations	0.934 [0.926; 0.942]	− 1.71	1,593,950
Extractions	0.804 [0.792; 0.816]	− 5.4	682,989
Radiographs	1.178 [1.168; 1.188]	4.08	1,735,041

Dependent variable: dummy variable for each treatment session of patient *i* at time *t* indicating whether it includes at least one procedure of the particular service basket or not. Model includes patient-level fixed effects. Confidence intervals are denoted in brackets

Appendix Tables A4 and A5 provide results of the robustness checks, comparing the intervention year with the post-intervention year. Odds ratios (OR) indicate a lower utilization in the reform period compared to the post-reform year (OR < 1) or a higher utilization compared to post-reform (OR > 1). For both age groups, the share of sessions including any preventive service was significantly lower in the reform period compared to the post-reform year (− 7.82%; OR 0.729 [95% CI 0.727; 0.732] and − 3.35%; OR 0.874 [95% CI 0.870; 0.879]). With regard to non-preventive services, the results were heterogenous. Whereas relative utilization of extractions (4.88%; OR 1.216 [95% CI 1.204; 1.228]) and radiographs (0.64; OR 1.026 [95% CI 1.022; 1.030]) for adults was higher during the reform period, the share of sessions including direct restorations (− 2.28%; OR 0.913 [95% CI 0.905; 0.920]) and radiographs (− 1.13%; OR 0.956 [95% CI 0.948; 0.964]) for children and adolescents was smaller during the intervention compared to the post-reform year.

Estimates for short-run OOP price elasticities of demand at a population level are depicted in Table 7. Under *α* = 0.01, all estimates are statistically significant and point towards a relatively inelastic demand near to 0 for preventive oral health care services. This is shown for both age groups under evaluation. The most elastic preventive services were preventive exams and oral hygiene advice for adults with an OOP price elasticity of demand of − 0.022 [95% CI − 0.023; − 0.021]. A price elasticity of − 0.022 means that a 1% increase in prices is associated with a 0.022% decrease in demand. Relatively higher elasticities are also observed for extractions and radiographs in the adult population with 0.061 [95% CI 0.046; 0.076] and 0.099 [95% CI 0.092; 0.106], respectively. Note that these elasticities showed a positive sign, suggesting an increase in demand with an increase in price. In general, estimates pointed towards more inelastic demand for children and adolescents than for adults. This is especially reflected in estimates for preventive exams and oral hygiene advice: whereas the elasticity for children and adolescents is − 0.002 [95% CI: − 0.003; − 0.002]; demand in the adult population was − 0.022 [95% CI − 0.023; − 0.021]. In the younger age group, we also observed slightly positive short-run price elasticities of demand for fissure sealants (0.001 [95% CI 0.000; 0.001]), extractions (0.004 [95% CI 0.002; 0.006]) and radiographs (0.012 [95% CI 0.011; 0.013]).

**Table 7:** OOP price elasticity of demand with provider fixed effects

Intervention	OOP price elasticity of demand [95% CI]	
	Age group 0–17	Age group 18+
Prev. exams and oral hygiene advice	– 0.002 [– 0.003; – 0.002]	– 0.022 [– 0.023; – 0.021]
Dental cleanings	– 0.008 [– 0.009; – 0.008]	– 0.002 [– 0.003; – 0.002]
Fissure sealants	0.001 [0.000; 0.001]	NA
Fluoride applications	– 0.001 [– 0.002; – 0.001]	NA
Direct restorations	– 0.009 [– 0.010; – 0.008]	0.001 [– 0.001; 0.003]
Extractions	0.004 [0.002; 0.006]	0.061 [0.046; 0.076]
Radiographs	0.012 [0.011; 0.013]	0.099 [0.092; 0.106]

Dependent variable: number of treatment sessions including the respective treatment provided by provider ${\text{p}}$ at time $t$. Model includes provider-level fixed effects. Confidence intervals are denoted in brackets

## Discussion

Little is known about how price liberalization in dental care affects prices of different services, price elasticities of demand, and, ultimately, utilization patterns. Relying on expansive claim-level insurance data, we evaluated the 2012 “Dutch-free market experiment” and analyzed the potentially distortive effects of the experiment on price and service utilization. We found substantial increases in patients’ OOP expenditures for dental services following the liberalization with differences in increases between services. Price liberalization affected the composition of treatment sessions, leading to a decreased share of general preventive exams. Our study is the first to show how price liberalization changes OOP prices in the dental care market and affects utilization patterns, especially with regard to preventive services.

Descriptive results showed price increases for all services under evaluation following the experiment’s introduction. A general price increase is in line with official evaluations by the NZa [32, 33]. It also supports the ideas regarding providers’ market power due a lack of information to the consumer, transaction costs of switching providers, and an overall limitation in supply [13]. It contrasts, however, with the results from the evaluation based on Swedish data that found negative effects of competition on prices [7]. Service fees for preventive services increased by up to 20% following the reform and 45% for extractions, respectively. Different price increases, especially regarding examinations and extractions, are in line with the theory of different effects of competition on the first-stage and follow-on services [6, 7]. These results are in line with those from Sweden. However, similarly to the study conducted in Norway [12], we are looking at a relatively short-time frame where market forces might not have fully settled at the time point of evaluation.

Due to maximum reimbursement rates, increasing service prices are also reflected in the OOP prices that patients face. For an insured population, this is the actual price of interest in the short run. We find consistent OOP price increases following the introduction of the FME for all service baskets under evaluation. For preventive care and care for children and adolescents, we observed a shift from full insurance coverage to a system with substantial patient co-payments. We also find substantial differences between services. In absolute terms, OOP price increases are much larger for follow-on services compared to first-stage services. This supports the theory that demand is more price sensitive for first-stage services and that asymmetric information is more pronounced for complex follow-on services.

We found substantial alterations in the composition of care utilized at an individual patient level following the introduction of the FME. For both age groups, the likelihood of a treatment session including any preventive service decreases by 5.3% for children and adolescents and by 3.4% for adults, respectively. This was mainly driven by decreases in the share of sessions including preventive examination and oral hygiene advice. We also observe slight increases for scaling and polishing in adults and fluoride applications in the younger age group. This could be explained by patients choosing clinical procedures instead of educative health-promoting approaches—such as the provision of oral hygiene advices—when confronted with rising OOP costs for preventive services.

The effects observed for non-preventive items were also heterogeneous. We observed a relative increase in the utilization of extractions for adults and an increase in the share of sessions including radiographs for children and adolescents. The changes in relative utilization among the analyzed treatment baskets indicate a shift from general check-up utilization towards more invasive follow-on treatments such as extractions. One possible reason for this shift could be that people, at least in the beginning of the reform, were postponing less time-sensitive utilization. Still, the observed changes in utilization patterns are in accordance with previous evidence on unintended offsets in medical care for chronically ill patients [17–19].

Our findings extend the evidence on undesired offsets for chronically ill to the general population in the context of dental care. Chair-side preventive services, such as regular check-ups, are effective measures to prevent oral disease [20, 21, 42–44]. Therefore, decreasing utilization of practice-based prevention, due to higher prices, may affect population’s oral health and treatment needs over the whole life cycle. Eventually, the resulting dental diseases could increase the cost burden through restorative care such as direct restorations, endodontic treatments, and replacements. Robustness checks comparing the year of the intervention with the post-reform year show that the effects on utilization of such an intervention can be reversed by returning to a fixed fee-for-services remuneration. Results of the robustness checks show a lower share of sessions including preventive services in the reform year compared to the post-reform period.

In addition to an isolated analysis of price and relative utilization changes, we examined short-run OOP price elasticities of demand for different services and age groups to examine patients’ OOP price sensitivity in the context of the experiment. In the absence of comprehensive claim-level individual data, previous analyses of demand’s reaction on price changes had to apply surrogate outcome measures such as expenditures per dental visit, which may suffer from a lack in accuracy and granularity [5, 14]. In general, we find relatively inelastic OOP price elasticities of demand close to zero. Especially with regard to prevention-oriented items of care, estimates are largely compatible to other estimates on oral health care services from insured populations evaluating long-term elasticities [15, 22, 23]. This contrasts with results from the RAND health insurance experiment, which suggest that elasticity may be much higher shortly after changes in prices [45]. We also found small differences in elasticities for children and adults but without a clear direction. Therefore, we cannot confirm findings suggesting less elastic demand for children’s oral health care [46, 47].

We found varying price elasticities for different services especially with regard to preventive check-ups and restorative services. In addition to the observation on service price development, this finding supports the existence of different price sensitivities for first-contact and follow-on services due to different levels of asymmetric information [6, 24]. Most remarkably, we observe small put positive price elasticities of demand for some non-preventive services, which means that demand increases in price for those items. Since we used actual utilization as a proxy for demand, these observations emphasize short-term market distortions following the experiment’s introduction that led to shift in the composition of utilization towards non-preventive services.

Our study faces a number of limitations. Due to substantial changes in treatment codes by a concurrent change in the fee schedule at the time of the experiment, evaluation of diagnostic and restorative items was limited to a set of comparable measures to avoid bias through inaccuracies in procedure harmonization. Our analysis was solely based on data from an insured population. Patients without insurance for dental care had to pay the complete provider fee out-of-pocket and, therefore, faced a higher increase in cost burden. Previous studies suggest that demand for oral health care services is more elastic in uninsured populations [22]. Theory would suggest, however, that these people shifted their relative utilization towards more prevention [16].

For the insured population, changes in OOP expenditures following the reform are a function of maximum compensation rates of the insurance and prices charged by the dentist. Since those maximum rates were largely based on previous regulated prices, it is difficult to separate out market power mechanisms from justified price adjustments due to previous imperfections in price regulations. Also note that a large proportion of the Dutch population has a dental care insurance (persons under the age of 18 years via the Health Insurance Act; many persons aged 18 years or older have complementary dental coverage). Along these lines, our findings could be interpreted in the sense of a lower bound estimate for the price elasticities of demand for dental care. It could be argued that, in a population with lower extents of dental coverage, patients might respond stronger to price changes, because they would then face larger OOP expenditures for dental care.

Another limitation of our study is that we cannot differentiate between adults having different levels of additional insurance coverage. Especially, the effects on relative utilization might differ for people with more extensive coverage as compared to those with more basic coverage. Similarly, we are not able to assess how the reform impacted people from different socio-economic backgrounds. The short duration of the experiment also means that market forces were not able settle and all results have to be understood as short-run effects.

Due to the differences in fee schedules, we were also unable to assess the impact of the FME on overall dental care utilization. We would expect higher OOP expenditures to lead to lower utilization of dental care services overall, all other things being equal. Within the limitations of our study, one potential interpretation of our findings could be that, rather than a welfare loss to society due to underutilization of preventive services, price liberalization of dental services may have resulted in a welfare loss due to overutilization of curative services. However, the findings of our study should be interpreted with caution, because our data do not lend themselves for definite conclusions about potential over- or underutilization.

Originally, the intention of the experiment on price liberalization was to improve quality, contain cost, promote entrepreneurship to lower regional scarcities, and improve market dissemination of product innovations [28]. A liberal market can serve as an instrument to reach these goals; however, several criteria must be met. Among these are equal bargaining power of supply and demand, no market access barriers, and transparency about price, content, and quality of the product.

Our results show that price liberalization of dental care came along with increasing service prices and substantial distortions in the composition of dental care demanded. This led to an unintended relative decrease in utilization of check-ups and increased financial burden of seeking oral health care. Observed patterns in price developments following the experiment’s introduction suggest that the FME was not capable to induce competition among suppliers. In fact, providers were able to raise prices due to specific market characteristics that avert equal bargaining power between supply and demand. Varying price sensitivities of patients for different services (expressed through OOP price elasticities of demand) point towards information asymmetries between patients and providers. Providers of dental care were able to exploit these asymmetries, since patients were not capable of assessing the quality and necessity of service to a sufficient extent due to insufficient transparency and knowledge.

Supply of providers was also fixed in the short run, since considerable market access barriers exist, starting with a limited number of students that is admitted to study dentistry. Such regulation in supply may give providers bargaining power hampering a smooth functioning of a deregulated market and may enable the extraction of a monopoly rent [11]. A previous report by the Dutch Consumer Association suggests that dental care providers may not have engaged widely in competition: during the experiment, a majority of dentists evaluated were not willing to accept a new patient for one specific treatment [48]. Patients might thus not have been able to compare prices and quality as well as choosing a preferred provider, potentially implying increased transaction costs of switching providers.

Altogether, the Dutch oral health care market at the time of the experiment does not seem to have met the requirements necessary for a well-functioning oral health care market, which could theoretically have served as an instrument to improve quality, reduce costs, and promote dissemination of innovations. To be successful, the design and conduct of such or similar experiments should, therefore, be based on careful consideration of the specific characteristics of health care markets (uncertainties, externalities, and asymmetric information) and these also apply to dental care. In addition, other approaches such as more performance-based financing models might also provide opportunities for improvements in dental care system design [49–51]. Moreover, policy options that increase demand-sided bargaining power can be considered in this context. Those may be an effective tool as shown by third-party payers’ reimbursement rates. In the US, the general level and increases over time of third-party payers’ reimbursement rates are substantially lower than the fees set by dentists themselves [52]. Promoting demand-sided bargaining power may be able to reduce the price effects induced through the presence of asymmetric information.

Our results show that careful analysis and monitoring is warranted to adequately weigh the intended and unintended consequences of changes in the design of dental reimbursement systems.

## Electronic supplementary material

Below is the link to the electronic supplementary material.

Supplementary material 1 (DOCX 39 kb)

## Data Availability

Due to ownership of data by Achmea, primary data cannot be made accessible to the general public.

## Footnotes

1 The “Achmea Health Database” is the private database created by Achmea for research purposes. The authors received access to an extract from that database as part of the ADVOCATE EU project.
