# Supplementary material for: Once bitten, twice shy? Lessons learned from an experiment to liberalize price regulations for dental care
Source: Eur J Health Econ. 2019 Dec 31;21(3):425–36. doi: 10.1007/s10198-019-01145-z (PMC7188704; doi:10.1007/s10198-019-01145-z)
Supplement: Supplementary file 1 — Supplementary material 1 (DOCX 39 kb) [file 10198_2019_1145_MOESM1_ESM.docx]

**Appendix**

**Once bitten, twice shy? Lessons learned from an experiment to liberalize price regulations for dental care**

Table A1 Harmonization table

| **Treatment basket** | **2011** | | **2012** | | **2013** | |
| --- | --- | --- | --- | --- | --- | --- |
|  | **Code** | **Description** | **Code** | **Description** | **Code** | **Description** |
| **Preventive examination/ oral hygiene instructions** | C11 | **Periodic preventive examination (first one in the calendar year)** (Periodiek preventief onderzoek/ eerste in kalenderjaar) | A111 | **Periodic check-up** (Periodieke controle) | C11 | **Periodic check-up** (Periodieke controle) |
|  | C12 | **Periodic preventive examination (second and further in the calendar year)** Periodiek preventief onderzoek, tweede en volgende in hetzelfde kalenderjaar | C112 | **Standard preventive information/ instruction** (Preventieve voorlichting en/of instructie standaard) | C13 | **Problem-focused consultation** (Probleemgericht consult) |
|  | M31 | **determination of the plaque-score** (Plaque-score) | C114 | **expanded preventive information/ instruction** (Preventieve voorlichting en/of instructie uitgebreid) | M01 | **Preventive information/ intruction (**Preventieve voorlichting en/ of instructie) |
|  | M70 | **Comprehensive nutrition analysis** (Uitgebreide voedingsanalyse) | C124 | **Consulation for evaluation of prevention** (Consult voor evaluatie van preventie) | M02 | **Consulation for the evaluation of prevention** (Consult voor evaluatie van preventie) |
| **Professional Cleaning** | M50 | **Limited dental cleaning** (Gebitsreiniging (beperkt)) | C212 | **Standard dental cleaning** (Gebitsreiniging standaard) | M03 | **Dental cleaning** (Gebitsreiniging) |
|  | M55 | **Average dental cleaning** (Gebitsreiniging (gemiddeld)) | C214 | **Expanded dental cleaning** (Gebitsreiniging uitgebreid) |  |  |
|  | M59 | **Expanded dental cleaning**  (Gebitsreiniging (uitgebreid)) |  |  |  |  |
| **Fissure Sealants** | V30 | **Sealant (including etching), first tooth** (Sealing (Inclusief etsen), eerste element) | C511 | **Sealant per tooth**  (Sealen per element) | V30 | Sealant, first tooth  **(Sealen eerste element)** |
|  | V35 | **Sealant (including etching) Other teeth during the same session**  (Sealing (Inclusief etsen), volgende element in dezelfde zitting) |  |  | V35 | **Sealant, any further teeth in the same session**  (Sealen ieder volgend element in dezelfde zitting) |
| **Fluoride application** | M10 | **Fluoride application (Including previous oral cleansing): Method I** (Fluoride applicatie (Inclusief voorafgaande mondreiniging): Methode I) | C611 | **Fluoride application decidious teeth**  (Beslijpen en/of fluorideren melkelement) | M05 | **Fluoride application decidious teeth**  (Beslijpen en/of fluorideren melkelement) |
|  | M20 | **Fluoride application (Including previous oral cleansing): Method II**  (Fluoride applicatie (Inclusief voorafgaande mondreiniging): Methode II) | C811 | **fluoride treatment upper and lower jaw**  (Fluoridebehandeling boven- en ondergebit) | M10 | **Fluoride treatment method I**  (Fluoridebehandeling methode I) |
|  | M21 | **Fluoride application (Including previous oral cleansing): Method II, groupwise**  (Fluoride applicatie (Inclusief voorafgaande mondreiniging): Methode II, groepsgewijs) |  |  | M20 | **Fluoride treatment method I**  (Fluoridebehandeling methode II) |
| **Radiographs^1^** | X10 | **Intra-oral radiograph**  (Intra-orale foto (Per opname)) | A311 | **Create and assess a small radiograph**  (Maken en beoordelen kleine röntgenfoto) | X10 | **Small radiograph**  (Kleine röntgenfoto) |
|  | X21 | **Panoramic radiograph**  (Orthopantomogram) | A321 | **Create and assess a panoramic radiograph**  (Maken en beoordelen kaakoverzichtsfoto) | X21 | **Panoramic radiograph**  (Kaakoverzichtsfoto) |
|  | X22 | **Panoramic radiograph for implant purposes in the edentulous jaw**  (Orthopantomogram tb b. v. implantologie in the edentate kaak) | A324 | **Create and assess an radiograph of the skull**  (Maken en beoordelen schedelfoto) | X22 | **Panoramic radiograph for implant purposes in the edentulous jaw**  (Kaakoverzichtsfoto t.b.v. implantologie in de teandeloze kaak) |
|  | X24 | **Radiograph of the skull**  (Röntgenschedelprofielfoto) | A327 | **Create and assess a multi-dimensional radiograph of the jaw**  (Maken en beoordelen meer-dimensionale kaakfoto) | X24 | **radiograph of the skull**  (Schedelfoto) |
|  |  |  |  |  | X25 | **Create a multi-dimensional radiograph of the jaw**  (Maken meerdimensionale kaakfoto) |
|  |  |  |  |  | X26 | **Assess a multi-dimensional radiograph of the jaw**  (Beoordelen meerdimensionale kaakfoto) |
| **Extractions^1^** | H10 | **Extraction**  (Extractie) | J311 | **Extraction**  (Trekken tand of kies) | H35 | **Complicated extraction with mucoperiostal folding**  (Moeizaam trekken tand of kies, met mucoperiostale opklap) |
|  | H15 | **Any further extraction in the same session and the same quadrant**  (Volgende extractie in dezelfde zitting en zelfde kwadrant) | J315 | **Complicated extraction**  (Moeizaam trekken tand of kies) | H11 | **Extraction**  (Trekken tand of kies) |
|  | H30 | **Complicated extraction without mucoperiostal folding**  (Gecompliceerde extractie zonder mucoperiostale opklap) |  |  | H16 | **Any further extraction in the same session and the same quadrant**  (Trekken volgende tand of kies, in dezelfde zitting en hetzelfde kwadrant) |
|  | H35 | **Complicated extraction with mucoperiostal folding**  (Gecompliceerde extractie met mucoperiostale opklap) |  |  |  |  |
|  |  |  |  |  |  |  |
|  | *claimed in combination:* | |  |  | *claimed in combination:* | |
|  | H20 | **Suture, per aveolus**  (Hechten, per alveole) |  |  | H21 | **Cost of suture materials**  (Kosten hechtmateriaal) |
|  | H21 | **Cost of suture materials**  (Kosten hechtmateriaal) |  |  | H90 | **Preparation of practice space**  **for surgery**  (Voorbereiding praktijkruimte  ten behoeve van chirurgische verrichtingen) |
|  | H25 | **Comprehensive wound debridement** (Uitgebreid wondtoilet) |  |  |  |  |
|  | H90 | **Preparation of practice space**  **for surgery**  (Voorbereiding praktijkruimte  ten behoeve van chirurgische verrichtingen) |  |  |  |  |
| **Direct restorations** | V10 | **Pit filling**  (Pitvulling) | E111 | **One-surface restoration**  (Eénvlaksvulling) | V11 | **One-surface restoration**  (Eenvlaksrestauratie) |
|  | V11 | **One-surface restoration**  (Eenvlaksrestauratie) | E112 | **Two-surface restoration**  (Tweevlaksvulling) | V12 | **Two-surface restoration** (Tweevlaksrestauratie) |
|  | V12 | **Two-surface restoration**  (Tweevlaksrestauratie) | E113 | **Three-surface restoration**  (Drievlaksvulling) | V13 | **Three-surface restoration**  (Drievlaksrestauratie) |
|  | V13 | **Three-surface restoration**  (Drievlaksrestauratie) | E114 | **Four- or five-surface restoration**  (Vier- of vijfvlaksvulling) | V14 | **Crown of plastic (composite?) material**  (Kroon van plastisch materiaal) |
|  | V14 | **Crown of plastic (composite?) material**  (Kroon van plastisch materiaal) | E131 | **Construction of plastic material for extreme tooth erosion per element**  (Opbouw van plastisch materiaal bij extreme gebitsslijtage per element) |  |  |
|  |  |  | E411 | **Placing a root-canal pin**  (Plaatsen wortelkanaalpin) |  |  |
|  | *claimed in combination:* | |  |  | *claimed in combination:* | |
|  | V20 | **Etching in advance of placing composite restoration**  (Etsen ten behoeve van composiet) |  |  | V20 | **Etching in advance of placing composite restoration**  (Etsen ten behoeve van composietvulling) |
|  | V21 | **Etching in combination with etchable underlay**  (Etsen in combinatie met etsbare onderlaag) |  |  | V21 | **Etching in combination with etchable underlay in advance of placing composite restoration**  (Etsen in combinatie met etsbare onderlaag ten behoeve van composietvulling) |
|  | V50 | **Drying of a tooth by means of a rubber dam**  (Droogleggen van elementen door middel van cofferdam) |  |  | V50 | **Drying of a tooth by means of a rubber dam**  (Droogleggen van elementen door middel van cofferdam) |
|  | V60 | **Indirect pulp-capping**  (Indirecte pulpa-overkapping) |  |  | V60 | **Indirect pulp-capping**  (Indirecte pulpa-overkapping) |
|  | V70 | **Para-pulp pin**  (Parapulpaire stift) |  |  | V70 | **Para-pulp pin**  (Parapulpaire stift) |
|  | V80 | **Root-canal pin**  (Wortelkanaalstift) |  |  | V80 | **Root-canal pin**  (Wortelkanaalstift) |
|  | V85 | **Further root-canal pins in the same tooth**  (Elke volgende wortelkanaalstift in hetzelfde element) |  |  | V85 | **Further root-canal pins in the same tooth**  (Elke volgende wortelkanaalstift in hetzelfde element) |

^1^ without orthodontic codes

Table A2 Descriptive statistics: Service fees for age group 18+

|  | **Average (SD) service fees** | | |  | **Annual percentage change in service fees** | |
| --- | --- | --- | --- | --- | --- | --- |
|  | **2011** | **2012** | **2013** |  | **2012** | **2013** |
| **Prev. exams & oral hygiene advice** | 19.95 € (2.74) | 21.65 € (6.09) | 21.04 € (4.33) |  | +1.70 € | -0.61 € |
| **Dental cleanings** | 28.87 € (13.07) | 32.83 € (13.86) | 36.27 € (22.27) |  | +3.96 € | +3.44 € |
| **Direct restorations** | 84.54 € (57.59) | 104.74 € (65.49) | 87.62 € (57.51) |  | +20.2 € | -17.12 € |
| **Extractions** | 52.19 € (44.36) | 73.34 € (64.93) | 58.61 € (50.74) |  | +21.15 € | -14.73 € |
| **Radiographs** | 31.80 € (21.10) | 32.28 € (20.90) | 32.28 € (20.51) |  | +0.48 € | 0 € |

Table A3 Descriptive statistics: Service fees for age group 0-17

|  | **Average (SD) service fees** | | |  | **Annual percentage change in service fees** | |
| --- | --- | --- | --- | --- | --- | --- |
|  | **2011** | **2012** | **2013** |  | **2012** | **2013** |
| **Prev. exams & oral hygiene advice** | 20.76 € (5.05) | 23.84 € (9.72) | 23.82 € (21.47) |  | +3.08 € | -0.02 € |
| **Dental cleanings** | 22.20 € (11.23) | 26.53 € (10.36) | 23.54 € (15.92) |  | +4.33 € | -2.99 € |
| **Fissure sealants** | 61.95 € (43.04) | 69.66 € (55.63) | 63.26 € (43.23) |  | +7.71 € | -6.40 € |
| **Fluoride applications** | 23.27 € (2.68) | 24.14 € (5.08) | 24.80 € (5.36) |  | +0.87 € | +0.66 € |
| **Direct restorations** | 81.62 € (63.12) | 102.96 € (71.56) | 84.19 € (64.25) |  | +21.34 € | -18.77 € |
| **Extractions** | 40.09 € (28.64) | 58.26 € (38.26) | 55.34 € (30.51) |  | +18.17 € | -2.92 € |
| **Radiographs** | 35.15 € (20.05) | 41.31 € (28.32) | 35.94 € (19.96) |  | +6.16 € | -5.37 € |

Table A4 Robustness check: Logistic regression with patient fixed effects for age group 18+ (during vs. after reform)

| **Intervention** | **Odds ratio during vs. after reform [95%-CI]** | **Associated percentage change** | **Observations** |
| --- | --- | --- | --- |
| **Prev. exams & oral hygiene advice** | 0.836 [0.833; 0.839] | -4.47 % | 7,107,268 |
| **Scaling and polishing** | 0.850 [0.847; 0.853] | -4.05 % | 5,823,149 |
| ***Any preventive service*** | 0.729 [0.727; 0.732] | -7.82 % | 6,501,160 |
| **Direct restorations** | 0.993 [0.989; 0.996] | -1.87 % | 6,017,941 |
| **Extractions** | 1.216 [1.204; 1.228] | 4.88 % | 1,174,962 |
| **Radiographs** | 1.026 [1.022; 1.030] | 0.64 % | 6,046,835 |

**Note.** Dependent variable: dummy variable for each treatment session of patient *i* at time *t* indicating whether it includes at least one procedure of the particular service basket or not. Model includes patient-level fixed effects. Confidence intervals are denoted in brackets.

Table A5 Robustness check: Logistic regression with patient fixed effects for age group 0-17 (during vs. after reform)

| **Intervention** | **Odds ratio during vs. after reform [95%-CI]** | **Associated percentage change** | **Observations** |
| --- | --- | --- | --- |
| **Prev. exams & oral hygiene advice** | 0.882 [0.877; 0.887] | -3.14 % | 3,302,769 |
| **Scaling and polishing** | 0.600 [0.595; 0.604] | -12.5 % | 2,187,367 |
| **Fissure sealants** | 1.091 1.080; 1.102] | 2.18 % | 1,307,316 |
| **Fluoride applications** | 0.938 [0.932;0.944] | -1.60 % | 2,469,098 |
| ***Any preventive service*** | 0.874 [0.870;0.879] | -3.35 % | 3,185,803 |
| **Direct restorations** | 0.913 [0.905; 0.920] | -2.28 % | 1,627,775 |
| **Extractions** | NA |  |  |
| **Radiographs** | 0.956 [0.948; 0.964] | -1.13% | 1,835,368 |

**Note.** Dependent variable: dummy variable for each treatment session of patient *i* at time *t* indicating whether it includes at least one procedure of the particular service basket or not. Model includes patient-level fixed effects. Confidence intervals are denoted in brackets.
